# Correction: Epigallocatechin-3-Gallate (EGCG), a Green Tea Polyphenol, Stimulates Hepatic Autophagy and Lipid Clearance

**DOI:** 10.1371/journal.pone.0096884

**Published:** 2014-05-01

**Authors:** The PLOS ONE Staff

## Notice of Republication

This article was republished on April 10^th^, 2014, to correct errors throughout the manuscript that were introduced during the typesetting process. Please download this article again to view the correct version. The originally published, uncorrected article and the republished, corrected article are provided here for reference.

## Supporting Information

File S1 Originally published, uncorrected article.
(PDF)

File S2 Republished corrected article.
(PDF)
