# Supplementary figures and images for: Epigallocatechin-3-Gallate (EGCG), a Green Tea Polyphenol, Stimulates Hepatic Autophagy and Lipid Clearance
Source: PLoS One. 2014 Jan 29;9(1):e87161. doi: 10.1371/journal.pone.0087161 (PMC3906112; doi:10.1371/journal.pone.0087161)

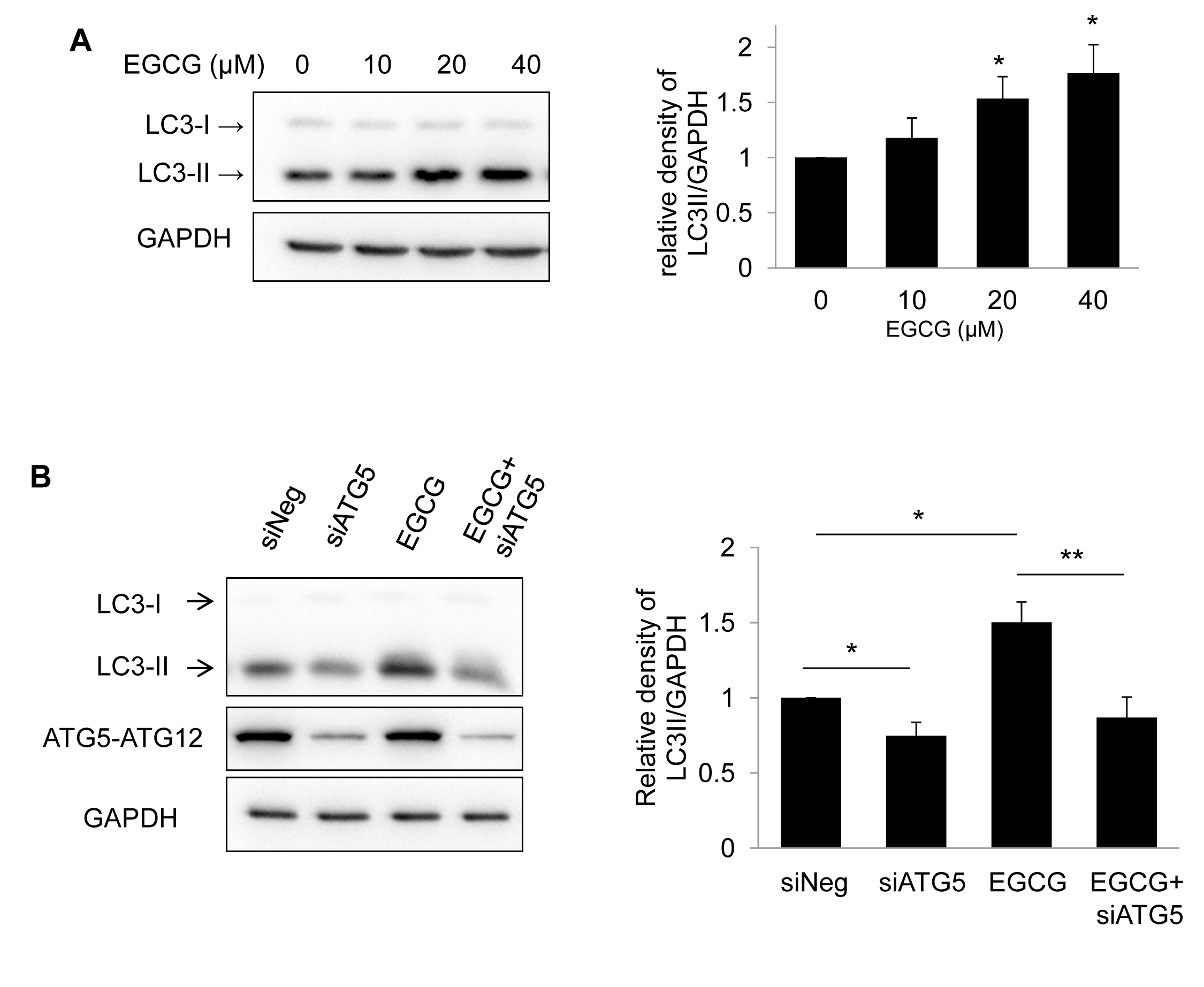

Supplement: Figure S1 — EGCG stimulates autophagy. (A) Immunoblot and densitometric analysis showing dose-response of LC3-II accumulation in Huh7 cells treated with indicated concentrations of EGCG for 24 hours. Bars represent the mean of the respective individual ratios±SD (n = 3). (B) Huh7 cells were transfected with negative or ATG5 siRNA and incubated for 24 hr. The cells were then treated without or with EGCG (40 µM) for 24 hr. (TIF) [file pone.0087161.s001.tif]

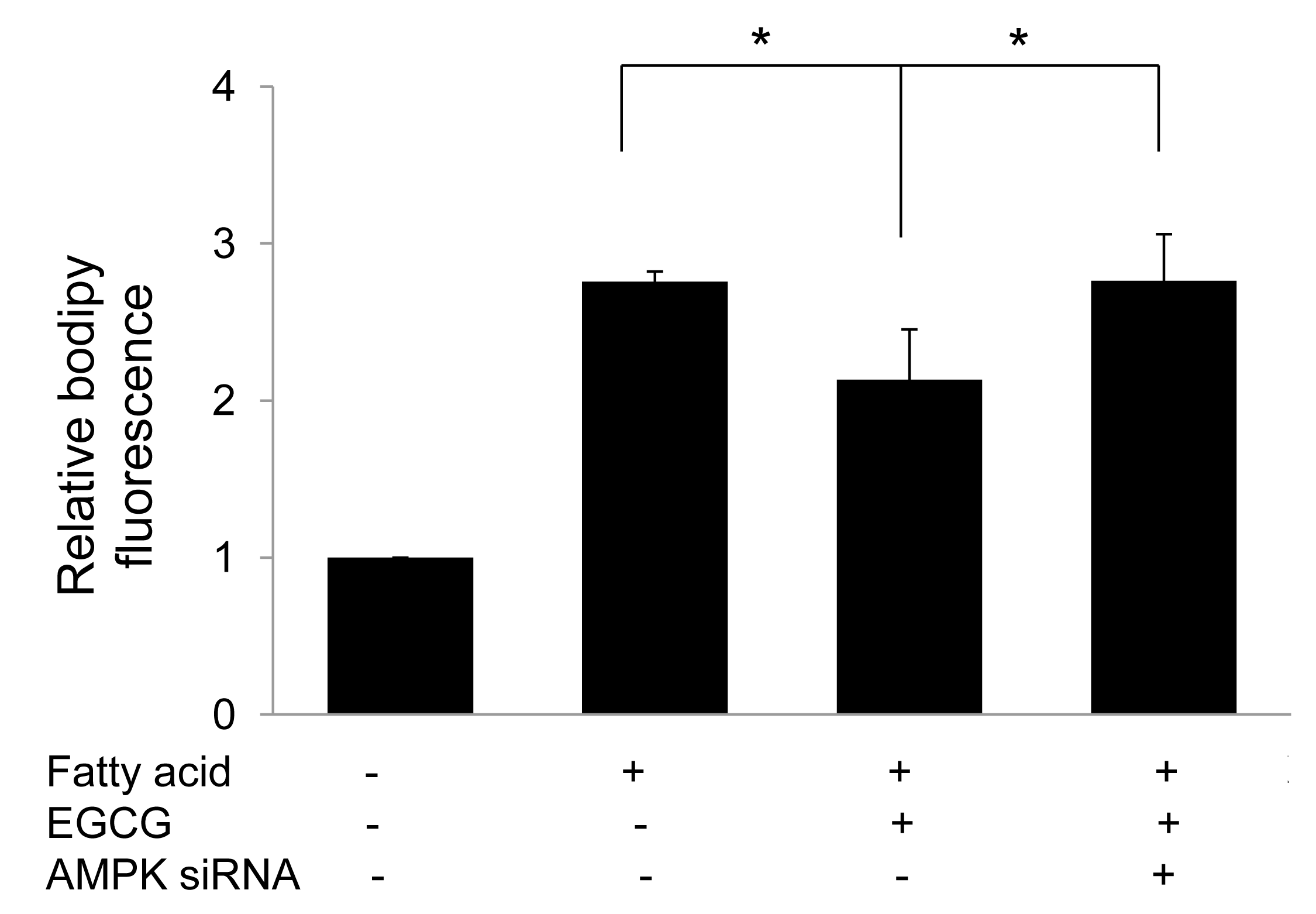

Supplement: Figure S2 — EGCG decreases intracellular lipid in an AMPK-denpendent manner. Huh7 cells were transfected with negative or AMPK siRNA and incubated for 24 hr. Cells were then pre-treated with 40 µM EGCG for 8 h, cotreated with fatty acid (0.1 mM palmitic acid and 0.2 mM oleic acid) and 40 µM EGCG for 16 hours, and post treated with 40 µM EGCG for 24 h. Lipid droplet was stained with bodipy 493/503, and measured by flow cytometry. Values are means±SD (n = 3). (TIF) [file pone.0087161.s002.tif]
