# Supplementary material for: Correction: Epigallocatechin-3-Gallate (EGCG), a Green Tea Polyphenol, Stimulates Hepatic Autophagy and Lipid Clearance
Source: PLoS One. 2014 May 1;9(5):e96884. doi: 10.1371/journal.pone.0096884 (PMC4006899; doi:10.1371/journal.pone.0096884)
Supplement: File S2 — Republished corrected article. (PDF) [file pone.0096884.s002.pdf]

OPEN ACCESS Freely available online

PLOS ONE

Epigallocatechin-3-Gallate (EGCG), a Green Tea Polyphenol, Stimulates Hepatic Autophagy and Lipid Clearance

Jin Zhou[1], Benjamin Livingston Farah[1], Rohit Anthony Sinha[1], Yajun Wu[2], Brijesh Kumar Singh[1], Boon-Huat Bay[2], Chung S. Yang[4], Paul Michael Yen[1,3]*

1 Program of Cardiovascular and Metabolic Disorders, Duke-NUS Graduate Medical School, Singapore, Singapore, **2** Department of Anatomy, Yong Loo Lin School of Medicine, National University of Singapore, Singapore, Singapore, **3** Sarah W. Stedman Nutrition and Metabolism Center, Departments of Medicine and Pharmacology and Cancer Biology, Duke University Medical Center, Durham, North Carolina, United States of America, **4** Departments of Chemical Biology, Ernest Mario School of Pharmacy, Rutgers, The State University of New Jersey, Piscataway, New Jersey, United States of America

Abstract

Epigallocatechin gallate (EGCG) is a major polyphenol in green tea that has been shown to have anti-inflammatory, anti-cancer, anti-steatotic effects on the liver. Autophagy also mediates similar effects; however, it is not currently known whether EGCG can regulate hepatic autophagy. Here, we show that EGCG increases hepatic autophagy by promoting the formation of autophagosomes, increasing lysosomal acidification, and stimulating autophagic flux in hepatic cells and *in vivo*. EGCG also increases phosphorylation of AMPK, one of the major regulators of autophagy. Importantly, siRNA knockdown of AMPK abrogated autophagy induced by EGCG. Interestingly, we observed lipid droplet within autophagosomes and autolysosomes and increased lipid clearance by EGCG, suggesting it promotes lipid metabolism by increasing autophagy. In mice fed with high-fat/western style diet (HFW; 60% energy as fat, reduced levels of calcium, vitamin D3, choline, folate, and fiber), EGCG treatment reduces hepatosteatosis and concomitantly increases autophagy. In summary, we have used genetic and pharmacological approaches to demonstrate EGCG induction of hepatic autophagy, and this may contribute to its beneficial effects in reducing hepatosteatosis and potentially some other pathological liver conditions.

Citation: Zhou J, Farah BL, Sinha RA, Wu Y, Singh BK, et al. (2014) Epigallocatechin-3-Gallate (EGCG), a Green Tea Polyphenol, Stimulates Hepatic Autophagy and Lipid Clearance. PLoS ONE 9(1): e87161. doi:10.1371/journal.pone.0087161

Editor: Hervé Guillou, INRA, France

Received September 9, 2013; **Accepted** December 18, 2013; **Published** January 29, 2014

Copyright: © 2014 Zhou et al. This is an open-access article distributed under the terms of the Creative Commons Attribution License, which permits unrestricted use, distribution, and reproduction in any medium, provided the original author and source are credited.

Funding: This work was supported by Duke-NUS Graduate Medical School Faculty Funds (PMY) sponsored by the Ministry of Health, Ministry of Education, and Ministry of Trade, Singapore and A*StaR. The funders had no role in study design, data collection and analysis, decision to publish, or preparation of the manuript.

Competing Interests: The authors have declared that no competing interests exist

* E-mail: paul.yen@duke-nus.edu.sg

Introduction

Autophagy is a highly conserved cellular process in eukaryotic cells involved in protein, lipid, and organelle degradation via the lysosomal pathway. Autophagy begins with the formation of double-membranous structures called phagophores, which elongate and engulf portions of the cytoplasm to form autophagosomes. Subsequently, autophagosomes fuse with lysosomes to form autophagolysosomes, where the engulfed contents are degraded by acidic lysosomal hydrolases [1]. Autophagy is involved in cell growth, survival, development, and cell death. Impaired autophagic flux has been associated with pathologic neurologic, musculoskeletal, immunologic, cardiovascular, and hepatic conditions [2].

The liver is one of the most important metabolic organs, and is highly dependent on autophagy for both normal function and protection against hepatic diseases such as non-alcoholic fatty liver disease, viral hepatitis, and fibrotic disorders. During periods of starvation, autophagy degrades cytoplasmic components to produce amino acids and fatty acids that can be used to synthesize new proteins or generate ATP for cell survival [3]. Additionally, there is considerable evidence that impaired autophagy contributes to a number of common hepatic diseases, including tissue injury due to toxins, high-fat-diet, ischemia/reperfusion, and viral hepatitis, as well as hepatocellular carcinoma [4,5].

Epigallocatechin-3-gallate (EGCG) is the most abundant polyphenol in green tea and has been thought to be responsible for most of latter's therapeutic benefits. In particular, EGCG has anti-steaotic effects on the liver [6–9]. Autophagy also has been shown to be involved in these beneficial effects. Currently, it is not known whether EGCG regulates hepatic autophagy. Given both the importance of hepatic autophagy and the beneficial effect of EGCG on pathologic liver conditions, we investigated whether EGCG regulates autophagy and lipid clearance in the liver.

Materials and Methods

Reagents

EGCG, Acridine orange (AO) from Sigma-Aldrich (St Louis, MO, USA). Antibodies recognizing LC3, GAPDH, SQSTM1/P62, p-AMPK, p-ACC, AMPK, and ACC were purchased from Cell Signalling Technologies (Danvers, MA, USA), whereas antibodies recognizing β-actin, and HRP conjugated secondary antibodies recognizing mouse and rabbit IgG were purchased

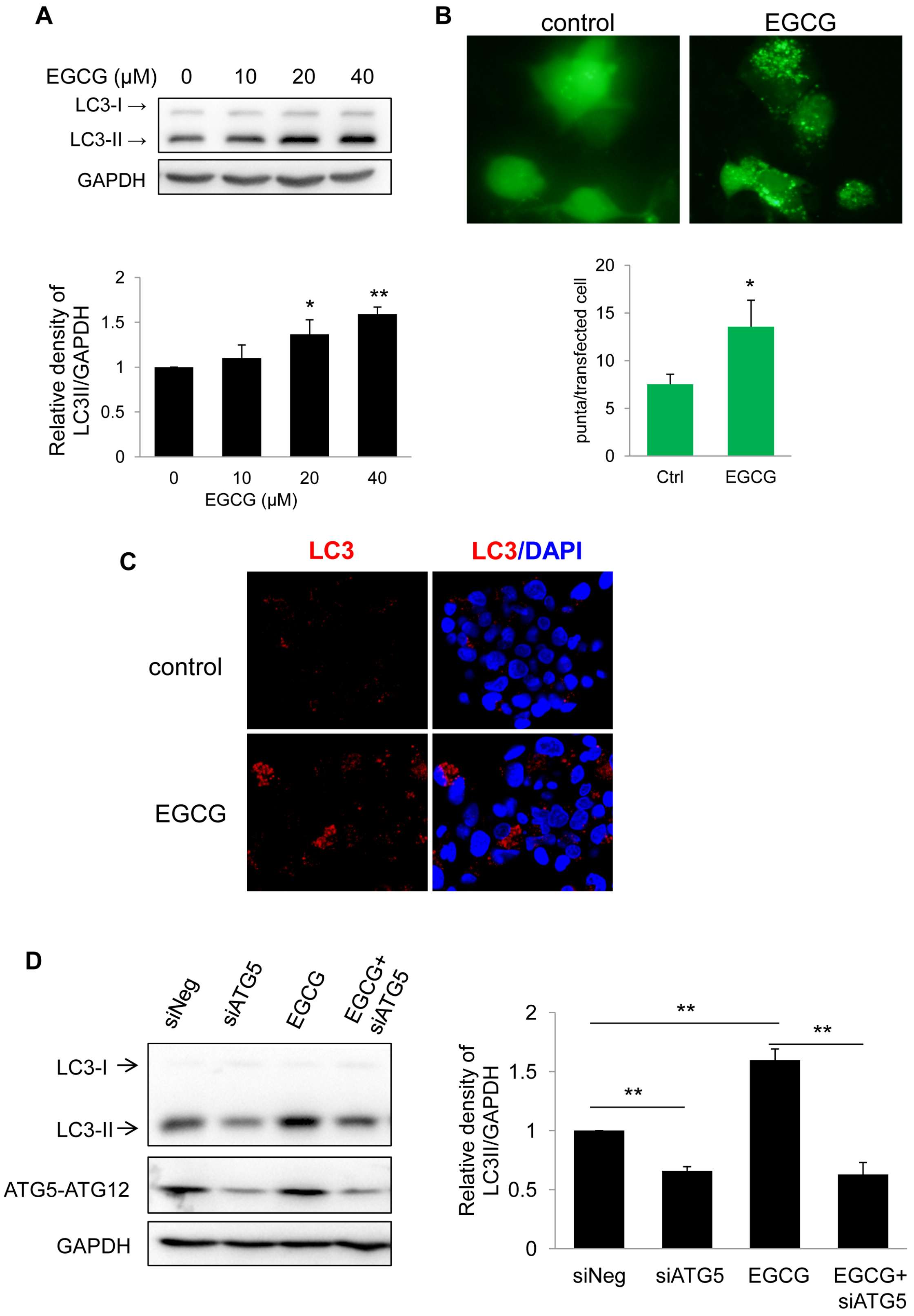

Figure 1. EGCG stimulates autophagy. (A) Immunoblot and densitometric analysis showing dose-response of LC3-II accumulation in HepG2 cells treated with indicated concentrations of EGCG for 24 hours. Bars represent the mean of the respective individual ratios±SD (n = 3). (B) A representative image and quantification of GFP-LC3 puncta in transfected HepG2 cells treated with or without 40 μM EGCG for 24 h. (C) LC3 immunostaining showing increased endogenous LC3-II puncta in HepG2 cells which were treated with 40 μM EGCG for 24 h. (D) HepG2 cells were transfected with negative or ATG5 siRNA and incubated for 24 hr. The cells were then treated without or with EGCG (40 μM) for another 24 hr.
doi:10.1371/journal.pone.0087161.g001

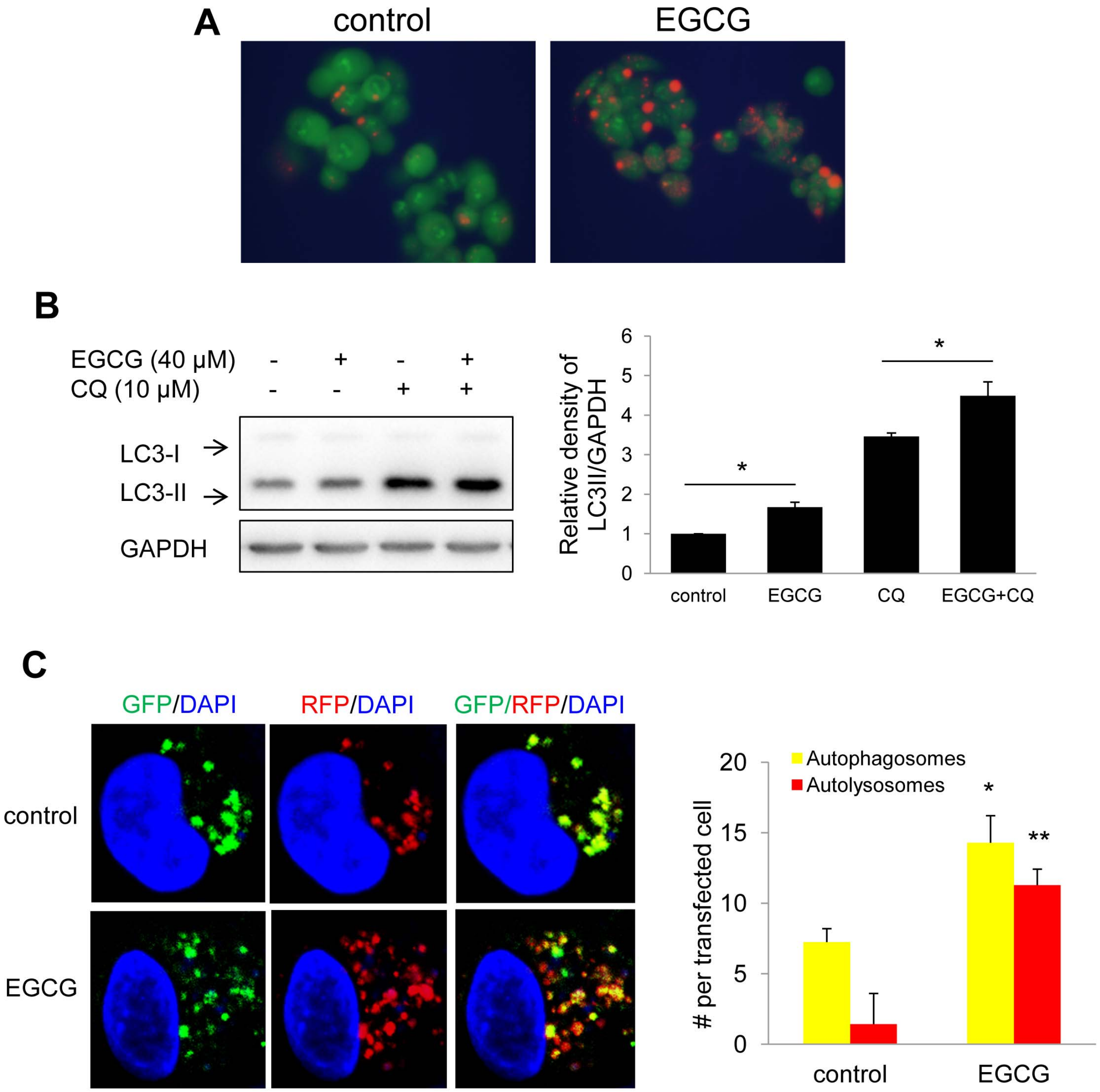

Figure 2. EGCG increases autophagy flux. (A) Acridine orange (AO) staining showing increase acidification in cells treated with 40 μM EGCG for 24 hours. (B) Evaluation of autophagic flux using lysosomal inhibitor chloroquine (CQ). HepG2 cells were pretreated with 40 μM EGCG for 20 hours followed by 6 hours co-treatment with 10 μM CQ. (C) Quantification and representative image of early autophagosomes (overlapping GFP+RFP puncta generating yellow puncta on overlay) shown as yellow bars and late autolysosomes (RFP puncta) shown as red bars after 24 hours of 40 μM EGCG treatment vs. non treated cells (Control) in tandem RFP/GFP-tagged LC3 plasmid transfected HepG2 cells. Bars represent the mean of the respective individual ratios±SE.
doi:10.1371/journal.pone.0087161.g002

from Santa Cruz Biotechnologies. Culture media and serum from Invitrogen (Madison, WI, USA). GFP-RFP-LC3 (tf-LC3) and eGFP-LC3 (Addgene plasmid 21073) plasmids were gifts from Prof. T. Yoshimori (Osaka University, Osaka, Japan) [9,10].

Cell Culture and Transfection

HepG2 and Huh7 cells were purchased from ATCC and maintained at 37°C in DMEM containing 10% FBS in a 5% CO_2 atmosphere. Primary mouse hepatocytes were isolated and culture using standard protocols.

For siRNA transfection, HepG2 cells were trypsinized, mixed with opti-MEM medium (Invitrogen) containing Lipofectamine RNAimax (Invitrogen) and ATG5 or control siRNA according to the manufacturer's recommendations. For cDNA transfection, GFP-RFP-LC3 (tf-LC3) and eGFP-LC3 plasmid was transfected into HepG2 cells using lipofectamine 2000 reagent (Invitrogen).

Western Blot Analysis

Proteins were separated by SDS–PAGE under reducing conditions and transferred to nitrocellulose membranes. Membranes were blocked with 5% nonfat milk in phosphate-buffered saline with 0.1% tween 20 (PBST). The blots were incubated overnight at 4°C with primary antibodies. Immunoblot analysis

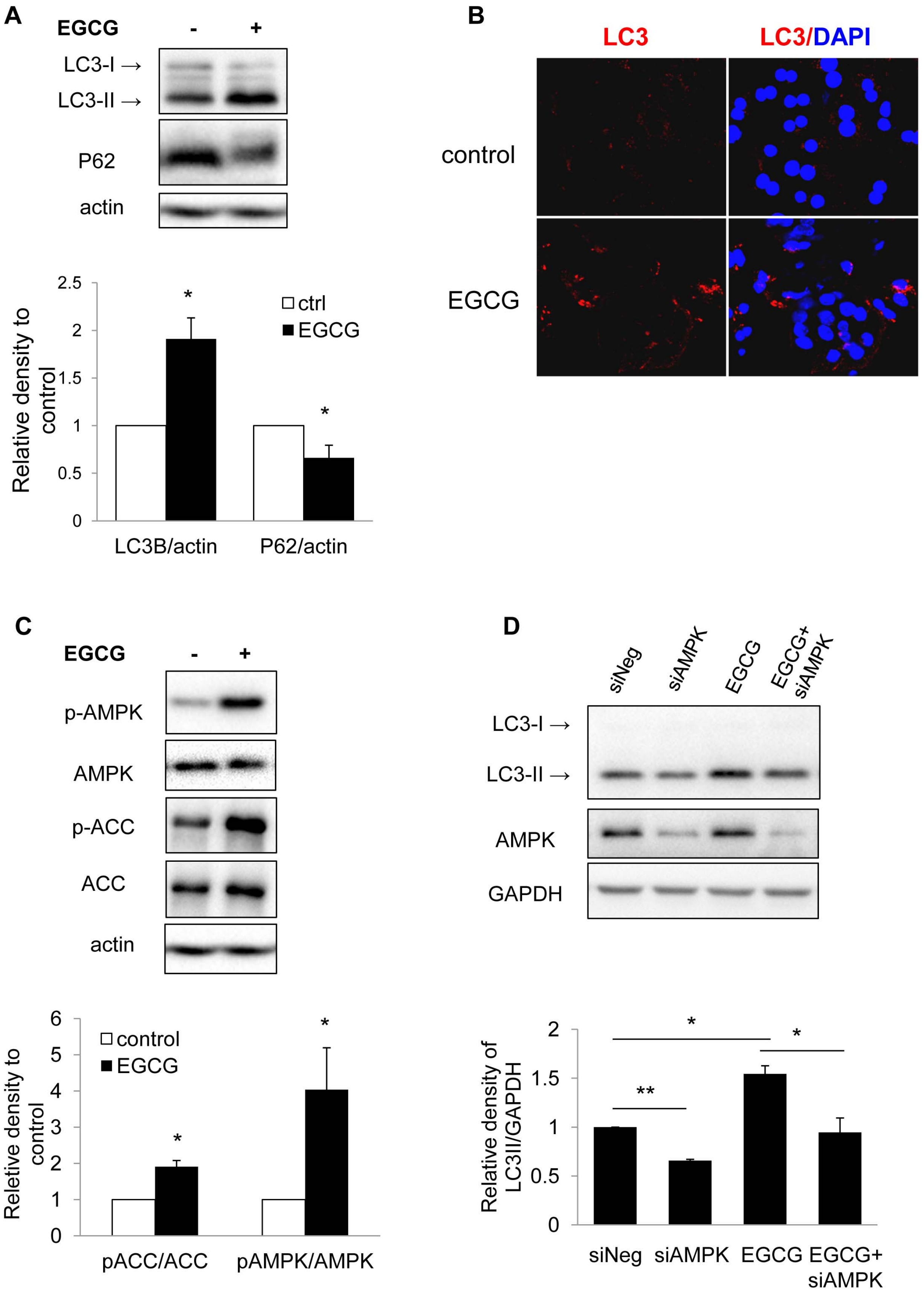

Figure 3. EGCG increase autophagy flux and AMPK phosphorylation in primary hepatocytes. (A) Immunoblots and densitometric analysis showing changes in LC3-II and P62 level in mouse primary hepatocytes cells treated with 40 μM EGCG for 24 h. Bars represent the mean of

the respective individual ratios±SD (n = 3). (B) LC3 immunostaining showing increased endogenous LC3-II puncta in primary mouse hepatocytes treated with 40 μM EGCG for 24 hrs. (C) Immunoblots and densitometric analysis showing changes in p-AMPK, and p-ACC level in mouse primary hepatocytes cells treated with 40 μM EGCG for 24 h. Bars represent the mean of the respective individual ratios±SD (n = 3). (D) HepG2 cells were transfected with negative or AMPK siRNA and incubated for 24 hr. The cells were then treated without or with EGCG (40 μM) for another 24 hr. Bars represent the mean of the respective individual ratios±SEM (n = 3).
doi:10.1371/journal.pone.0087161.g003

was performed using an enhanced chemiluminescence procedure (GE Healthcare).

Immunofluorescence Studies

For endogenous LC3-II puncta staining, cells were washed in PBS and then fixed with 4% paraformaldehyde for 15 minutes at room temperature. Fixed cells were washed with PBS, permeabilized in 100% methanol for 10 minutes at –20°C, washed in PBS, and blocked in blocking buffer for 1 hour at room temperature. Cells subsequently were incubated with anti-LC3 antibody overnight at 4°C. After 3 TBST washes, cells were incubated with Alexa Fluor–anti-rabbit antibody (Invitrogen) for 2 hours at room temperature and then washed 3 times in TBST. Coverslips were mounted on slides using Vectashield anti-fade reagent with 4′,6-diamidino-2-phenylindole (Invitrogen). Cells were observed under fluorescence microscope.

For ectopic eGFP-LC3 puncta, eGFP-LC3 plasmid was transfected into HepG2 cells with Lipofectamine 2000 Transfection Reagent (Invitrogen, Madison, WI, USA). Cells were observed under fluorescence microscope.

For autophagic flux analysis, tandem RFP/GFP-tagged LC3 plasmid was transfected into HepG2 cells with Lipofectamine 2000 Transfection Reagent (Invitrogen, Madison, WI, USA). Cells were visualized using LSM710 Carl Zeiss confocal microscope.

Acridine Orange Staining

Cells were grown on glass coverslips and treated with 40 μM EGCG for 24 h. Thereafter, the cells were incubated with either 1 μg/ml acridine orange (Sigma, St. Louis, MO, USA) for 15 min at 37°C followed by 3 PBS washes, and then immediately observed under fluorescence microscope.

Bodipy 493/503 Staining and Fat Measurement *in vitro*

Huh7 cells were pre-treated with 40 μM EGCG, cotreated with BSA-conjugated fatty acid (0.1 mM palmitic acid and 0.2 mM oleic acid) and 40 μM EGCG for 16 hours, followed by 24 hours treatment with 40 μM EGCG. Cells were incubated with the fluorescent dye BODIPY 493/503 (5 μg/ml, Invitrogen) for 10 min to stain intracellular lipid droplets. Subsequently, the cells were washed, resuspended in PBS, and analyzed (1×10^4 cells/measurement) using a Macsquant flow cytometer (Miltenyi Biotec).

Animal Models

Animal studies were conducted in accordance with the principles and procedures outlined in the National Institutes of Health Guide for the Care and Use of Laboratory Animals and were approved by the Institutional Animal Care and Use Committee (IACUC) at the Duke-National University of Singapore Graduate Medical School.

For acute EGCG administration, male C57BL/6 mice (8 weeks old) were obtained from NUSCARE and housed in hanging polycarbonate cages under a 12-hour light/12-hour dark cycle at 23°C with food and water available ad libitum. EGCG (25 mg/kg) were i.p injected daily for 3 days. Animals were sacrificed in CO2 chambers and livers were collected in liquid N2 and subsequently used for protein isolation.

For high-fat/Western-style diet and chronic EGCG treatment, male C57BL/6 mice ages 5 to 6 wks were purchased from Jackson Laboratories (Bar Harbor, ME). All animal experiments were carried out under protocol 91-024 approved by the Institutional Animal Care and Use Committee at Rutgers University (Piscataway, NJ). Mice were fed low-fat diet (LF; 10% energy as fat), high fat/Western-style diet (HFW; 60% energy as fat, reduced levels of calcium, vitamin D3, choline, folate, and fiber), or high-fat/Western-style plus EGCG diet (HFWE; HFW supplemented with 3.2 g EGCG/kg diet) for 17 wks. The dose of 3.2 g EGCG/kg diet inmice is equivalent to 10 cups of green tea (2 g tea leaves per cup) per day for an average person requiring 2000 kcal/d based on allometric scaling. Food intake and body weights were monitored weekly throughout the experiment [11].

Electron Microscopy

Fresh tissue was placed in fixative containing 2% paraformaldehyde and 3% gluteraldehyde in pH 7.4 phosphate buffer overnight at 4°C. Tissue was washed once in PBS, followed by post-fixation with 1% osmium tetroxide. Samples were dehydrated in washes with ascending concentrations of alcohol, followed by embedding in Araldite. Sections were cut and stained with uranyl acetate and lead citrate. Imaging was performed on Olympus EM208S transmission electron microscope.

Statistics

Cell culture experiments were performed in triplicates and repeated 3 independent times using matched controls. Results were expressed as mean±SD. Statistical significance was calculated using Student's t-test, taking $p<0.05$ as significant.

Results

EGCG Stimulates the Formation of Autophagosomes in HepG2 Cells

To investigate the effect of EGCG on hepatic autophagy, we first studied the well-characterized HepG2 human hepatoma cells, which retain many liver-specific metabolic functions [12]. LC3-II (microtubule-associated protein light chain 3-II), the phosphatidylethanolamine-conjugated form of LC3, is present in autophagosomes and thus is commonly used as a marker of autophagosome formation. Treatment with EGCG increased LC3-II formation in a dose-dependent manner in HepG2 cells (Fig. 1A). EGCG also induced LC3-II formation in the Huh7 human hepatoma cell line (Supplementary Fig. 1A). Cytoplasmic LC3 puncta are characteristic of autophagosomal membrane formation; thus, we examined EGCG induction of LC3 puncta by ectopic expression of eGFP-LC3 plasmid in HepG2 cells. As shown in Fig. 1B, eGFP-LC3 showed a diffuse distribution pattern in control cells, whereas it was arranged as cytoplasmic punctuate dots in EGCG-treated cells. We further confirmed these findings by immunofluorescence staining for endogenous LC3-II (Fig. 1C). To show that the EGCG-stimulated autophagosome formation occurred by an autophagy-dependent mechanism, we knocked down ATG5 in HepG2 and Huh7 cells by siRNA transfection, and then treated these cells with or without EGCG. Knockdown of ATG5 decreased EGCG-stimulated LC3-II in both HepG2 and

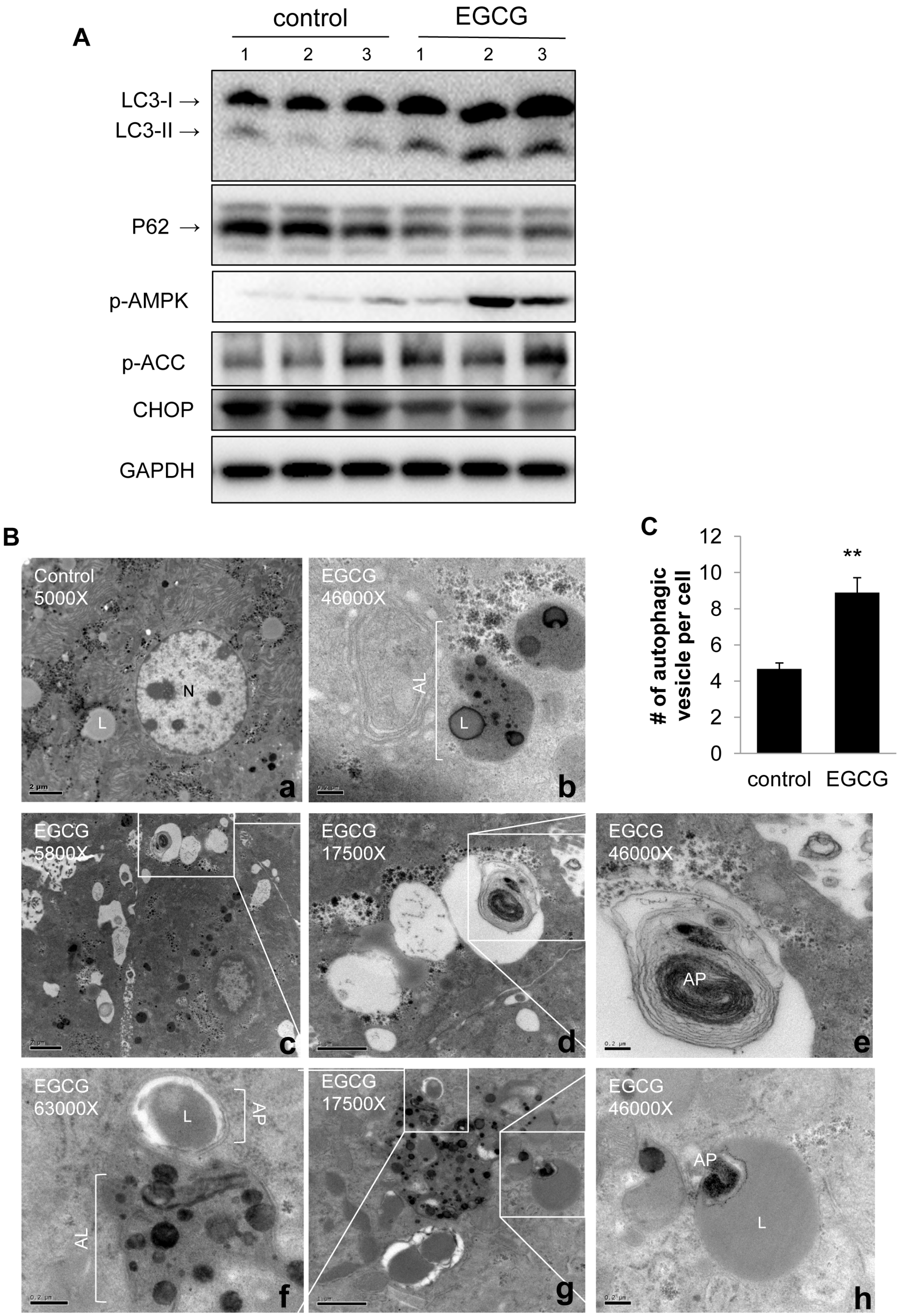

Figure 4. In vivo effects of EGCG on hepatic autophagy. (A) Immunoblots showing hepatic levels of LC3-II, p62, p-AMPK, p-ACC, and CHOP in EGCG treated mouse liver (i.p. administration of 25 mg/kg body weight EGCG for 3 days). (B) EM showing livers obtained from control (a) and EGCG

treated mice (b–h). (b) Aotolysosome with lipid droplet. (c-e) Autophagosome with different magnification. (f) Autophagosome with lipid droplet fusion with lysosome. (h) Autophagosome inside a large lipid droplet. L: lipid droplet; N: nuclear; AP: autophagosome; AL: autolysosomes. Scale bars: 2 μm (a and c); 1 μm (d and g); 0.2 μm (b, e, f and h). (C) Bar graphs showing Number of autophagic vesicles (including both autophagosome and autolysosomes) in control and EGCG treated mice liver based on EM micrograph images. Scoring was done by counting 5 different cells in 5 random fields per condition. Values are means±SE for 3 mice in each group.
doi:10.1371/journal.pone.0087161.g004

Huh7 cells (Fig. 1D and Supplementary Fig. 1B) suggesting that EGCG-stimulated autophagosome formation indeed occurred via an autophagy-dependent mechanism.

EGCG Increases Autophagic Flux in HepG2 Cells and Mouse Hepatocytes in Primary Culture

Autophagic flux progresses from autophagosome to autolysosome formation via fusion of autophagosomes with acidic lysosomes [13]. We assessed lysosomal activity using acridine orange, a lysosomotropic dye that emits orange fluorescence at low PH conditions [14]. We observed that EGCG-treated cells showed increased orange fluorescence indicating additional lysosomal acidification after EGCG treatment (Fig. 2A).

We next used two methods to assess whether EGCG facilitated autophagic flux. First, we compared the generation of LC3-II by EGCG alone or in combination with chloroquine (CQ). CQ neutralizes the lysosomal PH and blocks the degradation of the cargo in autophagosomes after fusion with lysosomes, and thereby blocks autophagic flux [14]. As shown in Fig. 2B, EGCG in combination with CQ showed increased levels of LC3-II compared with either EGCG or CQ alone, indicating increased autophagic flux is induced by EGCG. Second, we transfected tandem fluorescence RFP-GFP-LC3 (tf-LC3) plasmid into cells to demonstrate autophagic flux [10]. In this assay, GFP- and RFP-tagged to LC3 detects autophagosomes, whereas RFP detects only autolysosomes due to denaturation of GFP in the acidic environment of the autolysosome. Thus, in the overlaid images, yellow dots represent autophagosomes, and red dots represent autolysosomes. We observed that EGCG increased both autophagosome (yellow dots) and autolysosome (remaining red dots) formation in merged images (Fig. 2C) suggesting that fusion and protein degradation within the lysosome had occurred. Collectively, these results demonstrated increased autophagic flux after EGCG treatment.

We next investigated the effect of EGCG on hepatic autophagy by using primary mouse hepatocytes. EGCG treatment increased LC3-II levels (Fig. 3A) and LC3 puncta formation (Fig. 3B), confirming EGCG-induced autophagosome formation in primary hepatocytes. SQSTM1/p62 (p62) protein is an ubiquitin-binding scaffold protein that co-localizes with ubiquitinated protein aggregates in many proteinopathies of the liver. p62 accumulates when autophagy is inhibited, and decreases when autophagic flux occurs [15]. Immunoblotting showed significant reduction of p62 in EGCG-treated hepatocytes indicating by yet another method that there was increased autophagic flux (Fig. 3A).

EGCG Induces Autophagy by Stimulating AMPK Activity

AMPK promotes autophagy by directly phosphorylating mammalian autophagy-initiating kinase Ulk1, a homologue of yeast ATG1 [16]. EGCG treatment increased phosphorylation of AMPK (Fig. 3C), as well as the phosphorylation of the AMPK downstream target, acetyl-CoA carboxylase (ACC), indicating increased AMPK activity after EGCG treatment. Furthermore, knockdown of AMPK decreased EGCG-induced LC3-II in HepG2 cells (Fig. 3D), suggesting a critical role for AMPK in EGCG-stimulated autophagy.

EGCG Promotes Hepatic Autophagy *in vivo*

In order to demonstrate the effect of EGCG on hepatic autophagy *in vivo*, we injected C57BL/6 mice with EGCG (25 mg/kg) daily for 3 days. As shown in Fig. 4A, injection of EGCG increased hepatic LC3-II and decreased p62 protein levels in mouse livers, indicating increased autophagic flux. The phosphorylation of AMPK and ACC also was increased *in vivo* (Fig. 4A). Furthermore, CHOP, an endothelium reticulum (ER) stress marker [17], was decreased by EGCG.

Next, we observed the formation of autophagosomes and autolysosomes by electron microscopy. EGCG stimulated the formation of both autophagosome and autolysosomes (Fig. 4B and C). Interestingly, lipid was found inside double-membraned autophagosomes and autolysosomes (Fig. 4B, panels b and f), suggesting that EGCG induced-autophagy likely contributed to hepatic lipid metabolism.

EGCG-induces Lipid Clearance is Associated with Increase in Hepatic Autophagy both *in vitro* and *in vivo*

To futher understand the effect of EGCG on hepatic lipid metabolism, we first treated Huh7 cells with mixed fatty acid (palmitic and oleic acid) and examined the effect of EGCG on lipid clearance. Intracellular lipid content was determined by bodipy staining, followed by flow cytometry. As shown in Fig. 5A, intracellular lipid content was significantly decreased by EGCG. To determine whether autophagy induced by EGCG is directly involved in this process, we used ATG5 siRNA to block autophagosome formation. ATG5 knockdown significantly abolished EGCG-mediated reduction of intracellular lipid (Fig. 5B), and strongly suggested involvement of autophagy in the reduction of intracellular lipid. AMPK knockdown also blocked both EGCG-induced autophagy (Fig. 3D) and lipid clearance (Supplementary Fig. 2).

It was previously demonstrated that high-fat/western-style diet (HFW; 60% energy as fat, reduced levels of calcium, vitamin D3, choline, folate, and fiber) caused more severe hepatosteatosis and metabolic syndrome than high fat diet (HF, 60% energy as fat), and EGCG treatment significantly reduced liver triglyceride by 52% [11]. We performed immunoblots on the same hepatic samples and observed increased LC3-II in EGCG-treated HFW mice (Fig. 5C). Moreover, the ER stress marker CHOP was also reduced after EGCG treatment (Fig. 5C).

Discussion

Recently, there have been several reports suggesting that some of the beneficial effects of EGCG may be mediated by regulation of autophagy [18,19]. However, the effects of EGCG on autophagy seem to be tissue-specific. In macrophages, EGCG promotes autophagic degradation of endotoxin-induced HMGB1, a late lethal inflammatory factor [18]. The autophagy-promoting effect of EGCG also occurs in bovine aortic endothelial cells, and accounts for its reduction of lipid accumulation [19]. However, in human retinal pigment epithelial cells, EGCG reduces UVB light-induced retinal damage by down-regulation of autophagy [20]. Although EGCG also is protective against liver injury, it is not known whether it regulates hepatic autophagy to mediate these

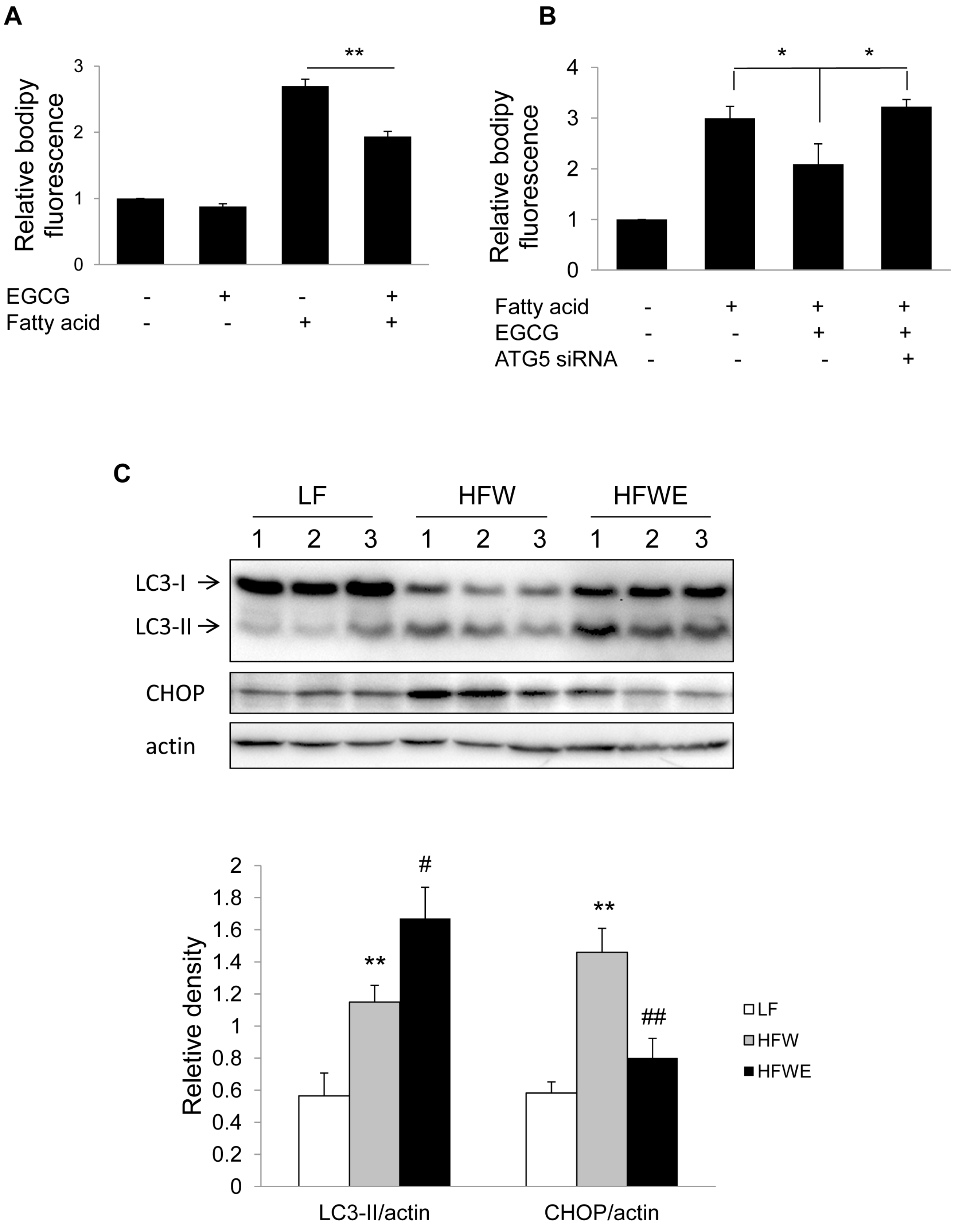

Figure 5. EGCG mediated fat clearance was associated with increased autophagy. (A) Huh7 cells were pre-treated with 40 μM EGCG, cotreated with fatty acid (0.1 mM palmitic acid and 0.2 mM oleic acid) and 40 μM EGCG for 16 hours, followed by 24 hours treatment with 40 μM EGCG. Lipid droplets was stained with bodipy 493/503, and measured by flow cytometry. Values are means±SD (n = 3). (B) Huh7 cells were transfected with negative or ATG5 siRNA and incubated for 24 hr, and followed by the same procedure as in panel A. (C) Immunoblots showing hepatic levels of LC3-II, and CHOP in mice fed with low fat (LF; 10% energy as fat), high fat/Western-style diet (HFW; 60% energy as fat, reduced levels of calcium, vitamin D3, choline, folate, and fiber), or high-fat/Western-style plus EGCG diet (HFWE; HFW supplemented with 3.2 g EGCG/kg diet) for 17 weeks. Densitometry values are means±SEM (n = 7). The significance between LF and HFW, HFW and HFWE was indicated by '*' and '#', respectively.
doi:10.1371/journal.pone.0087161.g005

effects. In our study, we provide several different lines of evidence to demonstrate that EGCG induces autophagy and autophagic flux in cultured hepatic cells and *in vivo*: First, EGCG induction of autophagosome formation was demonstrated by immunodetection of LC3-II, and visualization of ectopic and endogenous LC3-II puncta; second, EGCG increased lysosomal acidification; third, autophagic flux was demonstrated by co-treatment with CQ, tandem RFP-GFP-LC3 fluorescence, and immunodetection of p62; and fourth, EGCG-induced autophagy not only was observed in human hepatoma cell lines, but also in primary mouse hepatocytes and mouse liver. Thus, our study provides a better understanding of EGCG's actions on the liver, and strongly suggests that EGCG-induced autophagy may mediate some of its therapeutic effects.

Our electron micrographs showed co-localized lipid within the autophagosome and autolysosome compartments, demonstrating ingestion of cytosolic lipids by autophagosomes and their subsequent delivery to lysosomes. This autophagy-mediated degradation of intracellular lipid droplets plays an important role in hepatic fatty acid metabolism, and impaired autophagy leads to hepatosteatosis [21–24]. EGCG decreased lipid accumulation in several animal hepastosteatosis models, including hepastosteatosis induced by chronic high fat diet feeding [6], ethanol feeding [25], and acute ischemia/reperfusion injury [26]. Recently Kim *et al.* reported that EGCG decreased ectopic lipid accumulation by stimulating autophagic flux in bovine aortic endothelial cells [27]. We observed that EGCG significantly decreased hepatic triglyceride [11] and was associated with increased autophagy in mice on HFW diet for 17 weeks. Interestingly, acute administration of EGCG for three days increased co-localization of lipid inside autophagic vesicles suggesting that autophagy accounts for some of its actions to decrease hepatosteatosis. Furthermore, EGCG decreased intracellular lipid content in fatty acid treated cells in an autophagy-dependent mechanism. Altogether, our results show an essential role for EGCG induction of autophagy to reduce ectopic hepatic lipid accumulation caused by high fat diet. EGCG may modulate autophagy in other types of chronic and acute hepatic diseases. Last, it is noteworthy that EGCG also reduced hepatic CHOP levels *in vivo*, suggesting that it also may play a role in relieving ER stress in hepatic cells.

AMPK is a key mediator for the initial process of autophagy by stimulating the phosphorylation of ULK and formation of its protein complex with multiple autophagic proteins [16]. EGCG activates CaMKKβ/AMPK by stimulating Ca^{2+} release from ER stores [27]. Lin *et al.* reported that EGCG increased phosphorylation of AMPK and decreased lipid accumulation in cultured HepG2 cells [28]. We observed increased phosphorylation of AMPK and its downstream target ACC in cultured primary hepatocytes and *in vivo* after EGCG treatment. Furthermore, knockdown of AMPK by siRNA reduced EGCG-induced autophagy and lipid clearance in hepatic cells. Thus, AMPK activation likely is involved in the EGCG-induced autophagy in the liver.

In summary, we have used genetic and pharmacological approaches to demonstrate that EGCG stimulates autophagic flux in hepatic cells and *in vivo*. EGCG induction of autophagy decreases the lipid content in fat-loaded hepatic cells in culture, and likely does the same in livers of mice fed a HFW diet. These findings suggest that induction of autophagy by EGCG may reduce hepatosteatosis found in non-alcoholic fatty liver associated with obesity and diabetes, and may play an important role in reducing high fat diet-induced hepatosteatosis.

Supporting Information

Figure S1 EGCG stimulates autophagy. (A) Immunoblot and densitometric analysis showing dose-response of LC3-II accumulation in Huh7 cells treated with indicated concentrations of EGCG for 24 hours. Bars represent the mean of the respective individual ratios±SD (n = 3). (B) Huh7 cells were transfected with negative or ATG5 siRNA and incubated for 24 hr. The cells were then treated without or with EGCG (40 μM) for 24 hr.
(TIF)

Figure S2 EGCG decreases intracellular lipid in an AMPK-denpendent manner. Huh7 cells were transfected with negative or AMPK siRNA and incubated for 24 hr. Cells were then pre-treated with 40 μM EGCG for 8 h, cotreated with fatty acid (0.1 mM palmitic acid and 0.2 mM oleic acid) and 40 μM EGCG for 16 hours, and post treated with 40 μM EGCG for 24 h. Lipid droplet was stained with bodipy 493/503, and measured by flow cytometry. Values are means±SD (n = 3).
(TIF)

Acknowledgments

The authors would like to thank Ms. Anna Ba Liu (Departments of Chemical Biology, Ernest Mario School of Pharmacy, Rutgers, The State University of New Jersey) for assistance in animal work, Ms. Laura Martinez for assistance in obtaining primary hepatocytes, and Dr. Jianhong Ching, Dr. Chui Sun Yap, and Mr. Sherwin Ying Xie (Cardiovascular and Metabolic Disorders Program, Duke-NUS Graduate Medical School) for their helpful advice and constructive comments.

Author Contributions

Conceived and designed the experiments: JZ BLF RAS PMY. Performed the experiments: JZ BLF RAS BKS YW. Analyzed the data: JZ. Contributed reagents/materials/analysis tools: BB CSY PMY. Wrote the paper: JZ BLF RAS PMY.

References

1. Shintani T, Klionsky DJ (2004) Autophagy in health and disease: a double-edged sword. Science 306: 990–995.
2. Choi AM, Ryter SW, Levine B (2013) Autophagy in human health and disease. N Engl J Med 368: 651–662.
3. Kuma A, Hatano M, Matsui M, Yamamoto A, Nakaya H, et al. (2004) The role of autophagy during the early neonatal starvation period. Nature 432: 1032–1036.
4. Czaja MJ, Ding WX, Donohue TM, Friedman SL, Kim JS, et al. (2013) Functions of autophagy in normal and diseased liver. Autophagy 9: 1131–1158.
5. Kaushik S, Singh R, Cuervo AM (2010) Autophagic pathways and metabolic stress. Diabetes, Obesity and Metabolism 12: 4–14.
6. Bose M, Lambert JD, Ju J, Reuhl KR, Shapses SA, et al. (2008) The major green tea polyphenol, (−)-epigallocatechin-3-gallate, inhibits obesity, metabolic syndrome, and fatty liver disease in high-fat-fed mice. J Nutr 138: 1677–1683.
7. Tipoe GL, Leung TM, Liong EC, Lau TYH, Fung ML, et al. (2010) Epigallocatechin-3-gallate (EGCG) reduces liver inflammation, oxidative stress and fibrosis in carbon tetrachloride (CCl4)-induced liver injury in mice. Toxicology 273: 45–52.
8. Ciesek S, von Hahn T, Colpitts CC, Schang LM, Friesland M, et al. (2011) The green tea polyphenol, epigallocatechin-3-gallate, inhibits hepatitis C virus entry. Hepatology 54: 1947–1955.
9. Sun S-Y, Zhang Y, Yang N-D, Zhou F, Shen T, et al. (2012) (−)-Epigallocatechin-3-Gallate Induces Non-Apoptotic Cell Death in Human Cancer Cells via ROS-Mediated Lysosomal Membrane Permeabilization. PLoS ONE 7: e46749.
10. Kimura S, Noda T, Yoshimori T (2007) Dissection of the autophagosome maturation process by a novel reporter protein, tandem fluorescent-tagged LC3. Autophagy 3: 452–460.
11. Chen YK, Cheung C, Reuhl KR, Liu AB, Lee MJ, et al. (2011) Effects of green tea polyphenol (−)-epigallocatechin-3-gallate on newly developed high-fat/Western-style diet-induced obesity and metabolic syndrome in mice. J Agric Food Chem 59: 11862–11871.

12. Javitt NB (1990) Hep G2 cells as a resource for metabolic studies: lipoprotein, cholesterol, and bile acids. FASEB J 4: 161–168.
13. Hansen TE, Johansen T (2011) Following autophagy step by step. BMC Biol 9: 39.
14. Klionsky DJ, Abdalla FC, Abeliovich H, Abraham RT, Acevedo-Arozena A, et al. (2012) Guidelines for the use and interpretation of assays for monitoring autophagy. Autophagy 8: 445–544.
15. Bjorkoy G, Lamark T, Pankiv S, Overvatn A, Brech A, et al. (2009) Monitoring autophagic degradation of p62/SQSTM1. Methods Enzymol 452: 181–197.
16. Kim J, Kundu M, Viollet B, Guan KL (2011) AMPK and mTOR regulate autophagy through direct phosphorylation of Ulk1. Nat Cell Biol 13: 132–141.
17. Yang L, Li P, Fu S, Calay ES, Hotamisligil GS (2010) Defective hepatic autophagy in obesity promotes ER stress and causes insulin resistance. Cell Metab 11: 467–478.
18. Li W, Zhu S, Li J, Assa A, Jundoria A, et al. (2011) EGCG stimulates autophagy and reduces cytoplasmic HMGB1 levels in endotoxin-stimulated macrophages. Biochem Pharmacol 81: 1152–1163.
19. Kim HS, Montana V, Jang HJ, Parpura V, Kim JA (2013) Epigallocatechin Gallate (EGCG) Stimulates Autophagy in Vascular Endothelial Cells: A Potential Role for Reducing Lipid Accumulation. J Biol Chem 288: 22693–22705.
20. Li CP, Yao J, Tao ZF, Li XM, Jiang Q, et al. (2013) Epigallocatechin-gallate (EGCG) regulates autophagy in human retinal pigment epithelial cells: A potential role for reducing UVB light-induced retinal damage. Biochem Biophys Res Commun 438: 739–745.
21. Singh R, Kaushik S, Wang Y, Xiang Y, Novak I, et al. (2009) Autophagy regulates lipid metabolism. Nature 458: 1131–1135.
22. Sinha RA, Farah BL, Singh BK, Siddique MM, Li Y, et al. (2013) Caffeine stimulates hepatic lipid metabolism via autophagy-lysosomal pathway. Hepatology: n/a-n/a.
23. Takamura A, Komatsu M, Hara T, Sakamoto A, Kishi C, et al. (2011) Autophagy-deficient mice develop multiple liver tumors. Genes & Development 25: 795–800.
24. Sinha RA, You SH, Zhou J, Siddique MM, Bay BH, et al. (2012) Thyroid hormone stimulates hepatic lipid catabolism via activation of autophagy. J Clin Invest 122: 2428–2438.
25. Kaviarasan S, Viswanathan P, Ravichandran MK, Anuradha CV (2008) (−) Epigallocatechin Gallate (EGCG) Prevents Lipid Changes and Collagen Abnormalities in Chronic Ethanol-Fed Rats. Toxicol Mech Methods 18: 425–432.
26. Fiorini RN, Donovan JL, Rodwell D, Evans Z, Cheng G, et al. (2005) Short-term administration of (−)-epigallocatechin gallate reduces hepatic steatosis and protects against warm hepatic ischemia/reperfusion injury in steatotic mice. Liver Transplantation 11: 298–308.
27. Kim HS, Montana V, Jang HJ, Parpura V, Kim Ja (2013) Epigallocatechin Gallate (EGCG) Stimulates Autophagy in Vascular Endothelial Cells: A Potential Role for Reducing Lipid Accumulation. Journal of Biological Chemistry 288: 22693–22705.
28. Lin CL, Huang HC, Lin JK (2007) Theaflavins attenuate hepatic lipid accumulation through activating AMPK in human HepG2 cells. J Lipid Res 48: 2334–2343.
